# Effects of Freeze-Dried Burdock Root and Ultrasonic Treatment on the Growth and β-Glucosidase Activity of *Lactiplantibacillus plantarum* FEL112 in the Fermentation of Black Soymilk

**DOI:** 10.3390/foods15193563

**Published:** 2026-10-08

**Authors:** Chun-Wei Yang, Wen-Ling Hsiao, Chun-Yao Yang

**Affiliations:** Department of Food Science, Fu Jen Catholic University, No. 510, Zhongzheng Rd., Xinzhuang District, New Taipei City 242062, Taiwan; hanson0956@gmail.com (C.-W.Y.); 0920wenling@gmail.com (W.-L.H.)

**Keywords:** *Lactiplantibacillus plantarum*, black soymilk, burdock root, β-glucosidase, ultrasound

## Abstract

Burdock (*Arctium lappa* L.) root, rich in inulin and polyphenols, has physiological activities and can serve as a nutritional source for microorganisms. The aim of this study was to investigate the effects of freeze-dried burdock root powder (BP) and ultrasonic treatment (US) on the growth and β-glucosidase activity (β-GA) of *Lactiplantibacillus plantarum* FEL112 (*Lp*-FEL112) in the fermentation of black soymilk (BS). The viable cell counts and β-GA of *Lp*-FEL112 incubated in different amounts of BP in BS (x% BP/BS, *w*/*v*) without US or using US (40 and 120 kHz) for 1% BP/BS were evaluated. The total phenolic content (TPC), antioxidant activity, and isoflavones in the fermented product extract (FE) for 1% BP/BS were analyzed. The β-GA of *Lp*-FEL112 for 1% BP/BS without US at 24 h was 16.01 ± 1.88 U/mL, higher than those in 3% BP/BS and 7% BP/BS, and was enhanced to 18.62–26.86 U/mL using US. The antioxidant activities, TPC, and the isoflavone aglycones (daidzein and genistein) ratio in the FE of 1% BP/BS were improved with US. This study demonstrated that adding a limiting amount of BP to BS combined with US in the fermentation of BS by *Lp*-FEL112 could eliminate the need for an extraction step while achieving enhancement in the functionality of the fermented products.

## 1. Introduction

Burdock (*Arctium lappa* L.), a biennial herb plant belonging to the Asteraceae family and the genus Arctium, is an edible medicinal plant widely cultivated in many countries, especially in Asia and Europe, and has been used in the treatment of diuresis, hypoglycemia, eczema, and skin infection in traditional medicine [1,2]. The root of burdock contains a lot of bioactive substances, including chlorogenic acid (CGA) and caffeoylquinic acid derivatives, and inulin polysaccharides, and is considered to be a nutritious food with antioxidant, anti-inflammatory, anti-diabetic, and anti-obesity effects [3,4,5]. However, the nutritional effects in burdock root are also related to different processing methods, such as drying methods (hot-air drying, natural drying, sunlight drying, and vacuum freeze drying) [6]. Doi et al. (2015) reported that, in contrast to a raw burdock root diet, the feeding of 5% *Asperagillus awamori*-fermented burdock root diet effectively prevented hyperglycemia in mice caused by alloxan, indicating that the fermentation of burdock root could enhance the benefits for health [7].

Black soybean (*Glycine max* (L.), Merr.) is a good nutritional source of protein, carbohydrates, dietary fiber, lipids, vitamin B, vitamin C, vitamin E, and minerals (Na, K, P, and Fe); compared to yellow soybeans, black soybeans have a higher protein level, and abundant anthocyanins accumulate in the palisade layer of the seed coat’s epidermis [8]. Black soybean contains many antioxidants, including anthocyanin, saponins, and isoflavones, with physiological functions, such as anti-inflammation and anti-aging [9], and can be used to produce black soymilk (BS) beneficial to human health with significantly higher total phenolic content (TPC) and antioxidant capacity than yellow soymilk [10]. Isoflavone glycosides in BS can be biotransformed into their aglycones with higher bioavailability by β-glucosidase released from lactic acid bacteria (LAB) [11].

LAB can play a significant role in human health, such as regulating the immune system, inhibiting intestinal pathogens, lowering blood cholesterol, and preventing cancer [12]. However, the enzymatic hydrolysis rate of isoflavone glycosides to aglycones by LAB is usually hindered by semipermeable cellular membranes to retard the effective transport of β-glucosidase and converted products [13]. Yeo and Liong (2011) evaluated the effects of ultrasound on the growth of probiotics and bioconversion of isoflavones in prebiotic-soymilk, and indicated that the ultrasound-treated cells recovered from injury with the growth increased on fermentation in soymilk [13]. Gao et al. (2019) reported that the application of sonication significantly enhanced the release of taste compounds during moromi fermentation, and the possible mechanisms for such enhancement by sonication were the increased activities of enzymes and formation of more surface area and reaction sites in the sonicated moromis [14]. Therefore, to improve the enzymatic hydrolysis efficiency, ultrasound can be used as a means to stimulate the growth of LAB, so as to enhance the enzyme activities and the contact surface area during food fermentation [14,15].

In addition, the growth of LAB is substantially dependent on the nutrients in the culture medium [16]. When LAB encounter different sources of nutrients, it is crucial for LAB to efficiently metabolize carbohydrates and other carbon sources in the environment for survival [17]. In our previous study [18], Su et al. (2023) used the dried aqueous extract of burdock root (BWE), which would contain water-soluble polyphenols, glucose, fructose, and polysaccharide inulin, to serve as nutritional source to explore the impact of BWE on the survival of *Lactiplantibacillus plantarum* FEL112 via incubation in the medium of de Man, Rogosa and Sharpe (MRS) broth added with BWE or black soymilk (BS) added with BWE under ultrasonic stimulation [18].

In addition, in our previous studies [19,20], the inulin content in the dried burdock root powder of Yanagawarisou variety via ultrasound-assisted aqueous extraction (40 kHz, water solvent, and 10 min of extraction time) was about 42.89%/g-dried burdock root powder [19], and the CGA content via ultrasound-assisted ethanolic extraction (40 kHz, 95% ethanol solvent, and 120 min of extraction time) was about 818.74 µg/g-dried burdock root powder [20]. The information revealed that the freeze-dried burdock root powder (BP) of Yanagawarisou variety, which would contain the complete profile of bioactive compounds including water-soluble and less-hydrophilicity polyphenols, and other polysaccharides components, could be beneficial for the growth of *L. plantarum FEL112*.

Unlike the nutrient source from the medium of BWE (aqueous extract) in BS [18], the nutrient source provided by BP without undergoing an extraction process (not extract) in BS might affect substantially different growth of *L. plantarum* FEL112. Therefore, the aim of this study was to investigate the effects of supplementary nutrient source from BP added in BS and ultrasonic stimulation from various frequencies of ultrasound and treatment times on the growth and β-glucosidase activity of *L. plantarum* FEL112 during fermentation, and the total phenolic content (TPC), antioxidant activity, and isoflavone aglycones (daidzein and genistein) ratio in the fermented product were also evaluated.

## 2. Materials and Methods

### 2.1. Materials

The chemical reagents (±)-6-Hydroxy-2,5,7,8-tetramethylchromane-2-carboxylic acid (Trolox), *p*-nitrophenol β-D-glucopyranoside (*p*-NPG), Folin & Ciocalteu’s phenol reagent (2N), trifluoroacetic acid, *p*-nitrophenol, and ferric chloride were purchased from Merck KGaA (Darmstadt, Germany). *Lactobacilli* de Man, Rogosa and Sharpe (MRS) broth used as the base for the cultivation of *Lactobacilli* was purchased from Becton, Dickinson and Company (Franklin Lakes, NJ, USA). The chemicals daidzein, daidzin, genistein, and genistin used as the standards for the high-performance liquid chromatography (HPLC) analysis and other reagents were purchased from Merck KGaA (Darmstadt, Germany).

### 2.2. Activation of L. plantarum FEL112

The strain used in the fermentation of BP in BS was *L. plantarum* FEL112, which was isolated from Hakka sauerkraut [18]. *L. plantarum* FEL112 that had been preserved at −20 °C was activated, and the bacterial culture was inoculated at 1% into the sterilized MRS broth at 37 °C for 24 h. The prescribed procedure was performed again to complete the activation of *L. plantarum* FEL112.

### 2.3. Fermentation of BP in BS by L. plantarum FEL112 Without or with Ultrasonic Treatment

The black soybean (*G. max* (L.) Merr.) of Tainan No. 3 used to prepare black soymilk for fermentation was purchased from Shia Ying Farmers’ Associations (Tainan, Taiwan). A solid-to-liquid ratio of 1:8 (g/mL) was employed to soak the black soybean in distilled water for overnight (16–18 h). Then de-ionized water in a solid-to-liquid ratio of 1:8 (g/mL) was added in the mixture to agitate for 3 min. After that, the mixture was filtered, and the filtrate was sterilized at 121 °C for 20 min to obtain the BS for fermentation.

The burdock (*Arctium lappa* L.) roots of Yanagawarisou variety were purchased from Jiali District Farmers’ Association (Tainan, Taiwan). After a thorough wash, the purchased burdock roots were sliced, and freeze-dried by using HCS tray shelf freeze dryer (HC SCIENTIFIC & INSTRUMENT CO., New Taipei City, Taiwan) with the vacuum pump automatically starting at −40 °C (5 × 10^−3^ torr. of vacuum level) for three days. The freeze-dried slices were ground and screened by using 60-mesh sieves to obtain the freeze-dried burdock root powder (BP), and then preserved at 4 °C for use. The media for fermentation were prepared with different quantities (x% = 1, 3, and 7%) of BP added in BS (g-BP/mL-BS) (abbreviated as x% BP/BS), using BS without adding BP as the control, and sterilized at 121 °C for 20 min for use.

To evaluate the impact of various amounts of BP in BS on the growth and β-glucosidase activity of *L. plantarum* FEL112, the activated *L. plantarum* FEL112 was inoculated at 1% (about 7.7 log CFU/mL) into the medium of x% BP/BS to incubate at 37 °C for different times (0, 12, 24, 36, and 48 h) without ultrasonic treatment. The viable cell counts, pH value, and β-glucosidase activities were analyzed.

To evaluate the effect of ultrasonic treatment on growth and β-glucosidase activity of *L. plantarum* FEL112 incubated in 1% BP/BS medium, the activated *L. plantarum* FEL112 was inoculated at 1% into the medium of 1% BP/BS and then treated with ultrasound by using ultrasonic water bath (40 kHz/300 W or 120 kHz/300 W, 0.028 W/mL of power density) (Ultrasonicator, LEO-3002S, LEO-3002H, LEO Ultrasonic Co., Ltd., New Taipei City, Taiwan) controlled at 37 °C for 10 or 20 min, subsequently to incubate at 37 °C for different times (0, 12, 24, 36, and 48 h) [18]. The viable cell counts, pH values, and β-glucosidase activities were analyzed.

### 2.4. Analysis of Viable Cell Counts and β-Glucosidase Activity

For the determination of viable cell counts, the fermentation liquid was diluted in a ten-fold sequence to get an appropriate dilution ratio for the colonies counted in the range of 25–250 CFU/mL by using pour plate method.

The β-glucosidase activity was determined by following the method of Su et al. (2023) with a slight modification [18]. A 100-μL of *p*-nitrophenol β-D-glucopyranoside (*p*-NPG) (5 mM) was added into 1000 μL of the diluted fermentation liquid to react at 37 °C for 30 min. Then, 50 μL of sodium carbonate (1 M) was added to terminate the reaction, and the mixture was centrifuged with 12,000 rpm at 25 °C for 30 min for analysis using spectrophotometer (Hitachi, Ratio Beam Spectrophotometer U-5100, Tokyo, Japan) at 405 nm. The unit of β-glucosidase activity (U) was defined as the amount of β-glucosidase needed to release one nanomol of *p*-nitrophenol per mL of *p*-NPG per min.

### 2.5. Determination of Total Phenolic Content (TPC)

For the determination of TPC in the fermented product, the fermented product extract (FE) was prepared and freeze-dried. Then, a definite amount of FE was re-dissolved in 80% methanol to obtain the liquid sample for the analysis of TPC by following the method of Kujala et al. (2000) with a modification using gallic acid equivalent (GAE) [21]. A quantity of 250 μL of the liquid extract sample was mixed with 250 μL of Folin–Ciocalteu reagent to react at 25 °C for 5 min. Then, 500 μL of sodium carbonate solution (20%) was introduced into the mixture to react in the dark at 25 °C for 8 min. Subsequently, the mixture was centrifuged with 12,000 rpm for 8 min, and the absorbance of the supernatant was measured for TPC using spectrophotometer set at 730 nm. The TPC in FE was expressed as mg GAE/g-FE.

### 2.6. Determination of Ferric-Reducing Antioxidant Power (FRAP)

The antioxidant activity of the FE samples was determined with FRAP by using the Trolox equivalent (TE) and followed the method of Su et al. (2023) with a slight modification [18]. A 15-μL liquid sample of FE was mixed with 285 μL of FRAP reagent and 500 μL of de-ionized water to react at 37 °C for 4 min. The absorbance of the liquid sample was measured with a spectrophotometer set at 593 nm. The FRAP of the FE was expressed as mg TE/g-FE.

### 2.7. Analysis of Isoflavones

The liquid sample of FE was prepared and filtered through a 0.45-μm PTFE membrane for the HPLC analysis of daidzin, daidzein, genistin, and genistein contents in FE. The RP-18 column (5 μm, 250 mm × 4.6 mm, Kanto Chemical Co., Mightysil, Tokyo, Japan) and UV-VIS detector (Hitachi Chromaster 5420 UV-VIS detector, Hitachi, Ltd., Tokyo, Japan) at 260 nm were employed in the HPLC system, and the injection volume was 20 μL. The mobile phase was 0.1% trifluoroacetic acid (A) and 100% acetonitrile (B) with the solvent gradient set as 90% A at 0 to 35 min, 45% A at 35 to 45 min, and 90% A at 45 to 55 min.

### 2.8. Analysis of Surface Microstructure of L. plantarum FEL112

The surface microstructure of *L. plantarum* FEL112 was analyzed by referencing the method of Su et al. (2023) [18]. The fermented medium was centrifuged at 4 °C to separate the solid precipitates containing *L. plantarum FEL112*. After removing the supernatant, the solid sample was washed three times with 0.1 M phosphate buffer, and then completely immersed in 2.5% glutaraldehyde solution at room temperature overnight. After removing the supernatant by centrifugation, the sample was immersed in 1% osmium tetroxide solution at room temperature for 1.5 h, washed three times with 0.1 M phosphate buffer, and dehydrated by adding 35%, 50%, 60%, 70%, 80%, 90%, and 95% ethanol, respectively. The sample was centrifuged at room temperature and air-dried in a fume hood. The surface microstructure was analyzed with thermal field emission scanning electron microscope (FE-SEM) (JEOL, JSM-7800F, Tokyo, Japan).

### 2.9. Statistical Analysis

Three independent samples were used to perform the triplicate experiment for each condition, and the data were expressed as mean ± standard deviations (*n* = 3). The statistical analysis was carried out with one-way ANOVA by using IBM SPSS Statistics 20 (IBM Corp, Armonk, NY, USA), and the statistical differences within the same group were determined with Duncan’s multiple range test by setting *p* < 0.05 to be significantly different.

## 3. Results and Discussion

### 3.1. Effect of Various Amounts of BP in BS on the Growth of L. plantarum FEL112

**Figure 1:**
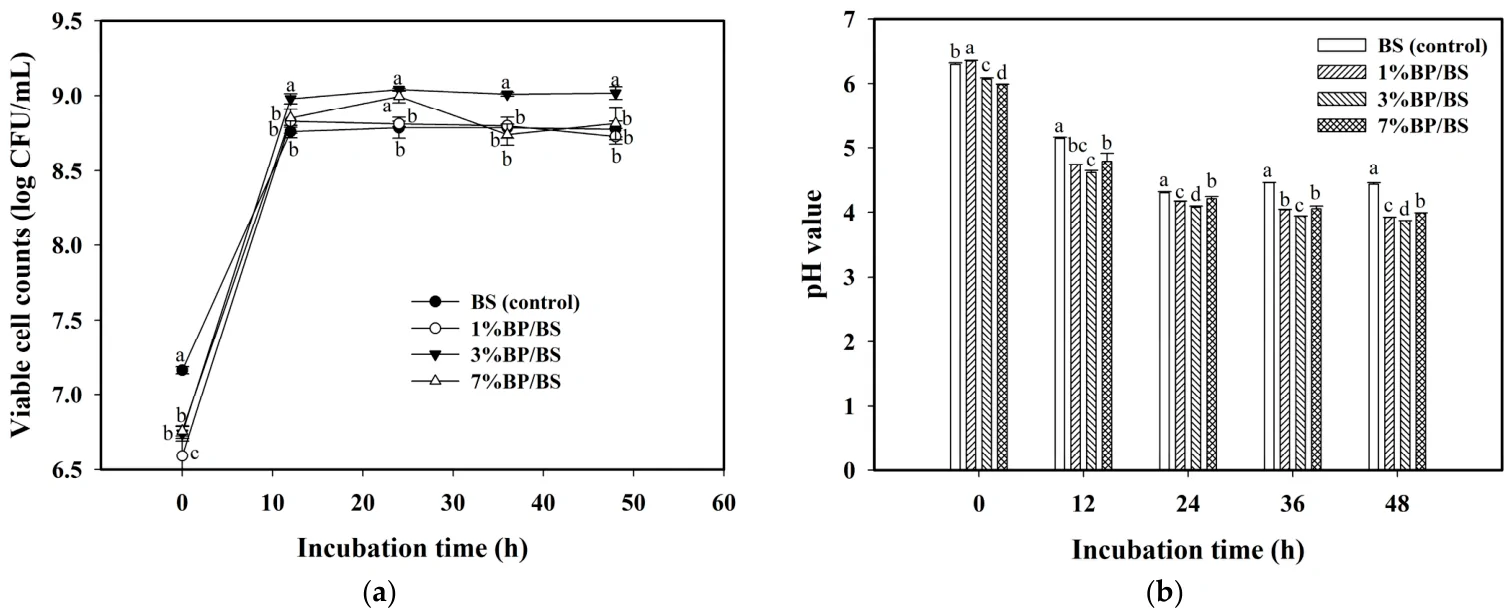
Comparison of the (**a**) viable cell counts and (**b**) pH values for *L. plantarum* FEL112 incubated in the medium of various amounts of freeze-dried burdock root powders (BP) added in black soymilk (BS) at 37 °C for 48 h. x% BP/BS: the medium of x% BP added in BS. Data were expressed as mean ± standard deviation (*n* = 3). Different lowercase letters at the same incubation time were significantly different (*p* < 0.05) by Duncan’s multiple range test.

The effect of supplementary nutrients from various amounts of BP (1, 3, and 7%) in BS without ultrasonic treatment on the growth of *L. plantarum* FEL112 was explored by using pure BS (without adding BP) medium as the control. The results of viable cell counts of *L. plantarum FEL112* and pH values of the media during 48 h of incubation time are shown in Figure 1a,b.

The viable cell counts of *L. plantarum* FEL112 were increased from 0 to 12 h for each medium, but were only slightly decreased from 12 to 48 h for BS (control), 1% BP/BS and 3% BP/BS media. In contrast for the 7% BP/BS medium, the viable cell counts decreased from 24 to 48 h. In addition, the viable cell counts were all higher than those for other tested media at each incubation time. This demonstrated that a suitable amount of BP could provide the enriched nutrients for the growth of *L. plantarum* FEL112, and too much solid BP (7%) with network structures added in BS medium might result in a larger steric hindrance for *L. plantarum* FEL112 to utilize the available nutrients so as to affect the growth of the strain.

Figure 1b displays the pH values for the tested media during 48 h of incubation. The results showed that the pH values for BS (control) medium at 12 to 48 h were all significantly higher than those for other media with the addition of BP. Moreover, the pH values of the 3% BP/BS medium were significantly lower than that those of 1% BP/BS and 7% BP/BS media. This indicated that the acidity in the 3% BP medium was relatively higher than those in other tested media during 12 to 48 h of incubation, possibly due to the expression of the higher viable cell counts and available nutrients from solid BP in 3% BP/BS. However, a lower pH value for 3% BP/BS might lead to reduced enzyme release and inhibited enzyme activities.

### 3.2. Effect of Various Amounts of BP in BS on β-Glucosidase Activity of L. plantarum FEL112

**Figure 2:**
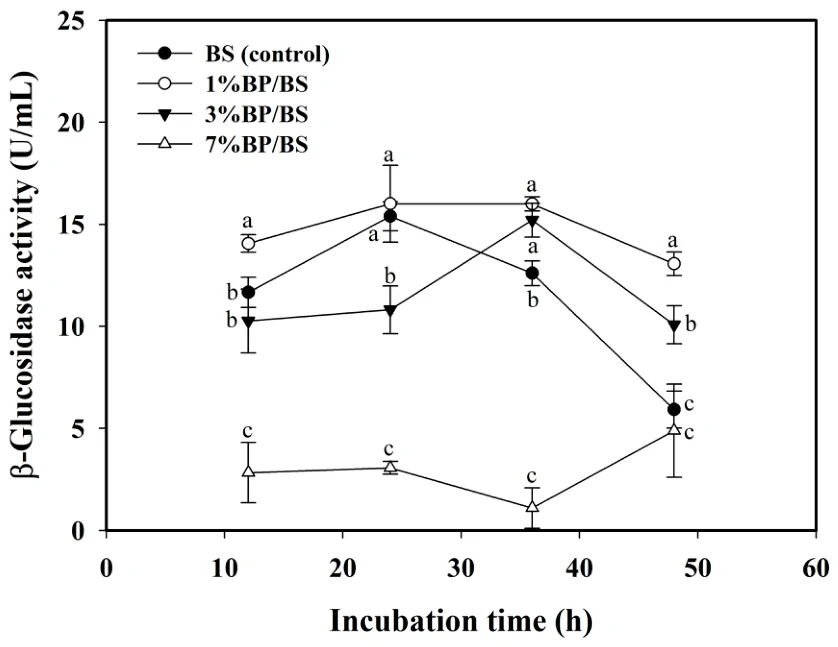
Effects of various amounts of freeze-dried burdock root powders (BP) added in black soymilk (BS) on the β-glucosidase activity of *L. plantarum* FEL112 at 37 °C for 48 h. x% BP/BS: the medium of x% BP added in BS. Data were expressed as mean ± standard deviation (*n* = 3). Different lowercase letters at the same incubation time were significantly different (*p* < 0.05) by Duncan’s multiple range test.

The BP contained a lot of polysaccharide inulin that could bind with caffeoylquinic acids to form the complex with some caffeoylquinic acids degraded during processing [19]. Thus, the fermentation level of BP and BS by *L. plantarum* FEL112 could be influenced by changing various amounts of BP in BS, and the acidity in the medium during fermentation could affect the availability of enzymes. Figure 2 displays the variations in β-glucosidase activity of *L. plantarum* FEL112 during 48 h of fermentation.

As shown in Figure 2, the β-glucosidase activities for the medium of 1% BP/BS were higher than those for the BS (control) and other media from 12 to 48 h of incubation time. This implied that the acidity level and steric hindrance effect existing in the medium would affect the β-glucosidase activity of the strain. Although the viable cell counts for 3% BP/BS medium were higher than those for 1% BP/BS, the higher acidity in 3% BP/BS than in 1% BP/BS would influence the release of β-glucosidase. Moreover, 3% BP/BS medium contained a relatively larger amount of solid BP with network structure than 1% BP/BS medium, such that the effect of steric hindrance on *L. plantarum* FEL112 to contact and utilize the nutrients from BP would be more impeded in 3% BP/BS than in 1% BP/BS [22]. Such a phenomenon was much more obvious for 7% BP/BS, where the β-glucosidase activity of *L. plantarum* FEL112 for 7% BP/BS medium was rather low during 48 h of incubation time, compared with that for 1% BP/BS. It was speculated that, using the solid BP (7%) with network structure added in BS, the steric hindrance for the strain to utilize the nutrients and the release of β-glucosidase could be much more impeded than 1%/BP/BS, leading to the lower β-glucosidase activity for 7% BP/BS. Considering the enzymatic hydrolysis efficiency in the fermentation of BS by adding different amounts of supplementary nutrients from solid BP, the level of β-glucosidase activity would be better to be used as the indicator for the selection of medium. Therefore, the 1% BP/BS medium was chosen to subsequently explore the effect of ultrasonic treatment on the enhancement of β-glucosidase activity of *L. plantarum* FEL112.

### 3.3. Growth of L. plantarum FEL112 Under Ultrasonic Stimulation for 1% BP in BS Medium

During fermentation with LAB, the bacteria usually encounter different stresses from the environment, including acid, cold, heat, salt, osmotic, and oxidative stresses [23]. In the fermentation process using ultrasound, the LAB will encounter the stimulation from ultrasonic irradiation [18]. In this study, after inoculating *L. plantarum FEL112* in 1% BP/BS medium, different levels of ultrasonic treatment on the incubation system before starting the fermentation were explored with 40 or 120 kHz of ultrasound irradiated for 10 or 20 min. When performing the ultrasonic treatment, the ultrasonic water bath was used to keep the temperature at 37 °C by controlling with ice to quickly diminish the possible temperature rise from ultrasonic irradiation. For convenience, the 1% BP/BS medium with ultrasonic treatment using x kHz of ultrasound for y min was denoted as 1% BP/BS (Ux-y min), and 1% BP/BS without ultrasound was used for comparison. The viable cell counts of *L. plantarum* FEL112 for various ultrasonic conditions and without ultrasound are shown in Table 1.

From Table 1, the viable cell counts of *L. plantarum* FEL112 for 1% BP/BS (U40-10 min), 1% BP/BS (U120-10 min), and 1% BP/BS (U120-20 min) were almost significantly higher than those for 1% BP/BS during 48 h of incubation, showing that a suitable ultrasonic treatment had positive impact on the growth of *L. plantarum* FEL112. Yeo and Liong (2011) indicated that the strain treated by ultrasound could recover from cell injury and showed enhanced growth in the fermentation of soymilk [13]. In this study, the ultrasonic treatment on the medium in the presence of the strain before starting fermentation not only had the impact on stimulating the growth of the strain but could also help loosen the network structure of solid BP, so as to improve the transport of ingredients and contact of the strain to the nutrients in the fermentation system. In addition, among the various conditions of ultrasonic treatment, 1% BP/BS (U120-10 min) would induce the strain *L. plantarum* FEL112 to have a longer stationary phase with higher viable cell counts at 36 and 48 h, showing that a relatively mild intensity of ultrasonic stimulation (120 kHz) would be valued in enhancing the growth of *L. plantarum* FEL112 in the fermentation of BS.

**Figure 3:**
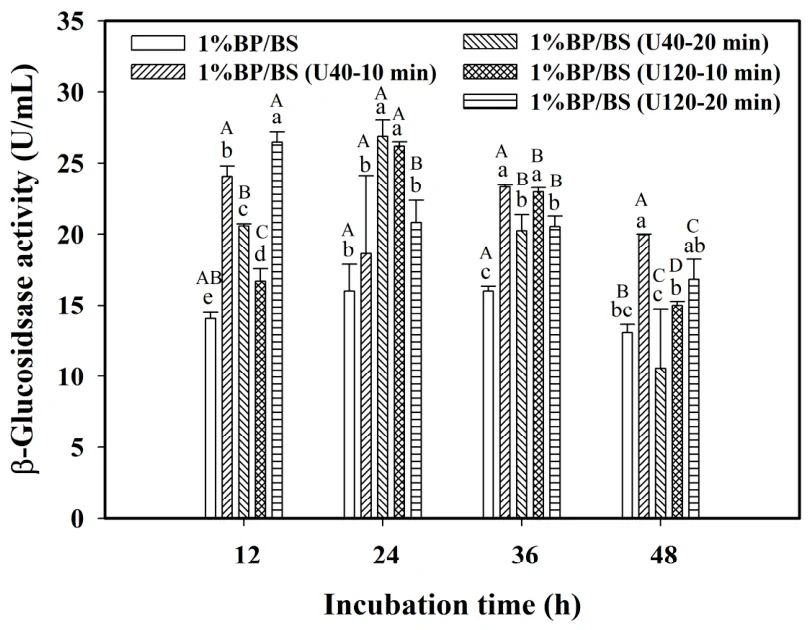
Comparison of the β-glucosidase activity of *L. plantarum* FEL112 incubated at 37 °C for 48 h in 1% BP/BS medium with or without ultrasonic treatment. 1% BP/BS: the medium of 1% freeze-dried burdock root powders (BP) added in black soymilk (BS) without ultrasonic treatment; 1% BP/BS (Ux-y min): the 1% BP/BS medium with ultrasonic treatment using x kHz of ultrasound for y min. Data were expressed as mean ± standard deviation (*n* = 3). Different lowercase letters at the same incubation time or different uppercase letters at the same medium condition were significantly different (*p* < 0.05) by Duncan’s multiple range test.

Figure 3 shows the β-glucosidase activities of *L. plantarum* FEL112 for 1% BP/BS (U40-10 min), 1% BP/BS (U40-20 min), 1% BP/BS (U120-10 min), 1% BP/BS (U120-20 min), and 1% BP/BS. In 12 h of incubation, the β-glucosidase activities of *L. plantarum* FEL112 in the 1% BP/BS medium with ultrasonic treatment were all significantly higher than those without ultrasound (14.06 ± 0.43 U/mL), and the highest β-glucosidase activity (26.46 ± 0.70 U/mL) was induced by U120-20 min with about 1.88-fold of that for 1% BP/BS without ultrasound. It was found that the β-glucosidase activities for 1% BP/BS without ultrasonic treatment were significantly the lowest during 48 h of incubation, demonstrating that the ultrasonic stimulation using both 40 and 120 kHz of frequency could effectively induce a positive impact on *L. plantarum* FEL112 to release a larger amount of β-glucosidase for utilizing the available nutrients in the medium.

In addition, after 24 h of incubation, the β-glucosidase activities for 1% BP/BS (U40-20 min) (26.86 ± 1.20 U/mL) and 1% BP/BS (U120-10 min) (26.18 ± 0.30 U/mL) were significantly higher than those for other ultrasonic conditions, while at 36 h, the β-glucosidase activities for 1% BP/BS (U40-10 min) (23.31 ± 0.14 U/mL) and 1% BP/BS (U120-10 min) (22.96 ± 0.30 U/mL) became to be significantly higher than those for other ultrasonic conditions. The results indicated that the higher intensity of ultrasound (40 kHz) irradiated for 10 min (1% BP/BS (U40-10 min)) induced the response of *L. plantarum* FEL112 to release β-glucosidase maintaining it at a rather steady manner with only a gradual decrease after 24 h of incubation. In addition, the highest β-glucosidase activity for various ultrasonic conditions occurred at different incubation times. Behzadnia et al. (2019) investigated the stimulation of biosurfactant production by *L. plantarum* ATCC 8014 in standard and modified MRS culture medium under various ultrasonic frequencies, and concluded that the microbial activity and the productivity of biological processes could be intensified by controlled ultrasound exposure and supplementation of culture media [23]. It was speculated that by using different intensities of ultrasonic stimulation on *L. plantarum* FEL112, the time needed for cell recovery from injury might be different, leading to the highest β-glucosidase activity being presented at different incubation times [13,23].

### 3.4. TPC and FRAP in the FE of 1% BP in BS Medium with Ultrasonic Treatment

**Figure 4:**
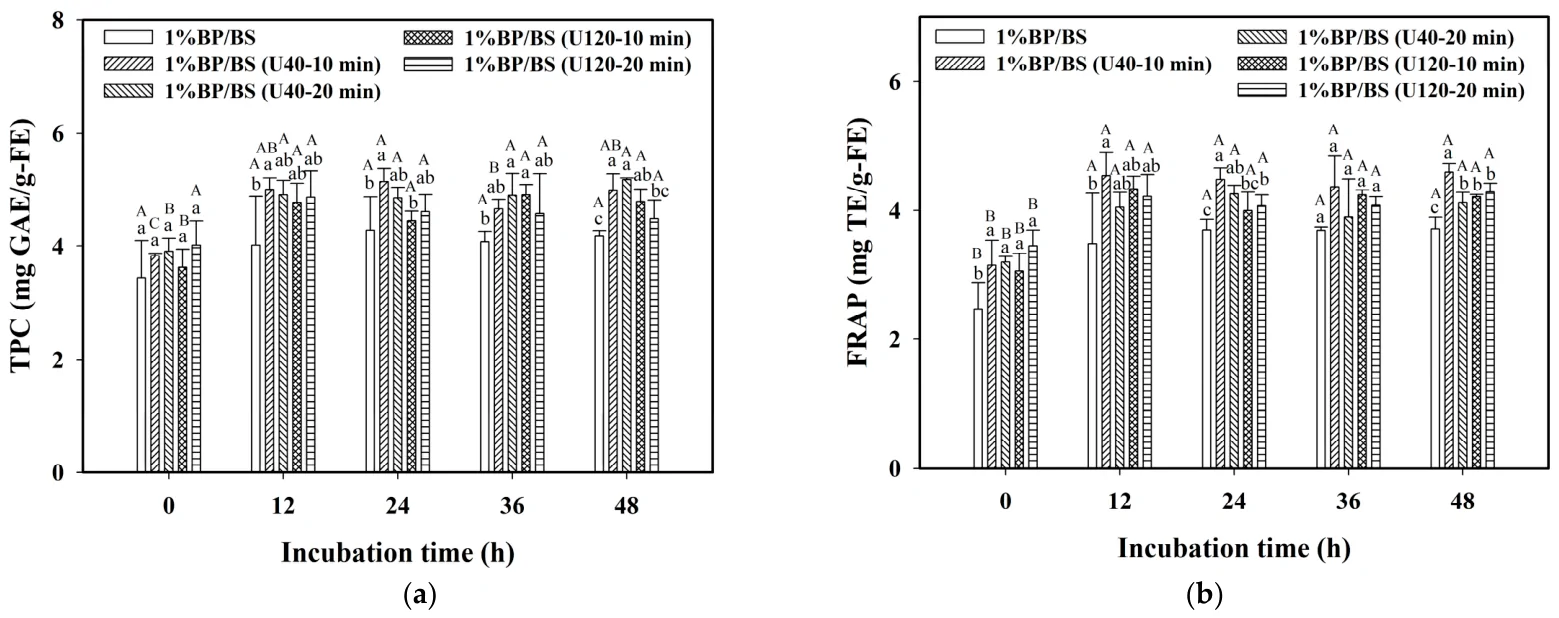
Variations of (**a**) total phenolic content (TPC) and (**b**) ferric-reducing antioxidant power (FRAP) of the dried fermented product extract (FE) for 1% BP/BS fermented by *L. plantarum* FEL112 with or without ultrasonic treatment at 37 °C for 48 h. 1% BP/BS: the medium of 1% freeze-dried burdock root powders (BP) added in black soymilk (BS); 1% BP/BS (Ux-y min): the 1% BP/BS medium with ultrasonic treatment using x kHz of ultrasound for y min. Data were expressed as mean ± standard deviation (*n* = 3). Different lowercase letters at the same incubation time or different uppercase letters at the same medium condition were significantly different (*p* < 0.05) by Duncan’s multiple range test.

The variations in TPC and FRAP in the FE during 48 h of fermentation by *L. plantarum* FEL112 under various conditions of ultrasonic treatment are shown in Figure 4a,b. The values of TPC at the initial time (0 h) in the FE with various conditions of ultrasonic treatment were all slightly higher than those without ultrasound (3.437 ± 0.658 mg GAE/g-FE), but with insignificant differences (*p* = 0.130). After 12 h of incubation time, the TPC in the FE for 1% BP/BS (U40-10 min) (4.996 ± 0.210 mg GAE/g-FE) was significantly higher than those for 1% BP/BS (4.015 ± 0.864 mg GAE/g-FE), and was slightly higher than those for 1% BP/BS (U40-20 min), 1% BP/BS (U120-10 min), and 1% BP/BS (U120-20 min), but with insignificant differences (*p* = 0.607). The TPC in the FE for 1% BP/BS (U40-10 min) at 24 h was the highest at 5.143 ± 0.228 mg GAE/g-FE, compared with those for other ultrasonic conditions. In contrast, at 48 h of incubation time, the TPC in the FE (4.987 ± 0.291 mg GAE/g-FE) for 1% BP/BS (U40-10 min) was insignificantly less than that for 1% BP/BS (U40-20 min) at 5.173 ± 0.030 mg GAE/g-FE, which was the highest in 48 h and greatly higher than those for 1% BP/BS (4.178 ± 0.088 mg GAE/g-FE). Liang et al. (2023) investigated the fermentation of five phenolic compounds by *Aspergillus oryzae*, *Lactobacillus plantarum* and *Saccharomyces cerevisiae*, respectively and reported that the glycosidic bonds in the phenolic compounds were hydrolyzed during fermentation, leading to the formation of additional phenolic metabolites in the fermented samples [24]. In this study, the results showed that using ultrasound of both 40 and 120 kHz to stimulate *L. plantarum* FEL112 before incubation could facilitate the enzymatic hydrolysis reaction of bioactive compounds in the 1% BP/BS medium, such that the TPC in the fermented products would be enhanced, especially with 40 kHz of high-intensity ultrasound [25].

As shown in Figure 4b, the results showed that the FRAP values in the period of 12 to 48 h of fermentation for the 1% BP/BS medium either with or without ultrasonic treatment were all significantly higher than those at the initial time (0 h). This demonstrated that the antioxidant activity of FRAP in the FE could be enhanced for 1% BP/BS medium during fermentation by *L. plantarum* FEL112 either with or without ultrasonic treatment, possibly due to the increase in the corresponding TPC after fermentation (Figure 4a) [26]. Furthermore, the FRAP could be maintained at almost steady values from 12 to 48 h for 1% BP/BS with 40 and 120 kHz of ultrasonic treatment, and the FRAP values for 1% BP/BS (U40-10 min) at 48 h of fermentation time were significantly higher than those for 1% BP/BS, 1% BP/BS (U40-20 min), 1% BP/BS (U120-10 min), and 1% BP/BS (U120-20 min), implying that treatment with a higher intensity of ultrasound (40 kHz) for a shorter time (10 min) was beneficial for producing a fermented product of BP in BS with higher TPC and antioxidant activity of FRAP.

### 3.5. Effect of Ultrasonic Treatment on Isoflavones in the FE of 1% BP in BS

The effect of ultrasonic treatment on the profile of isoflavone glycosides (daidzin and genistin) and their aglycones (daidzein and genistein) for the 1% BP/BS medium during fermentation by *L. plantarum* FEL112 was evaluated by using the weight percentage ratio of daidzein and genistein (AI) to sum of daidzin, genistin, daidzein, and genistein (TI) as the indicator. The results are shown in Table 2.

From Table 2, the results showed that for 1% BP/BS without ultrasonic treatment, the AI/TI ratios in the FE were 63.08% at 12 h and 74.48% at 48 h of fermentation time. By using ultrasonic treatment for the 1% BP/BS medium, the AI/TI ratios in FE were enhanced to 76.17% at 12 h and 81.67% at 48 h for 1% BP/BS (U40-10 min) and improved to 79.42% at 12 h and 81.79% at 48 h for 1% BP/BS (U120-10 min). Furthermore, the AI/TI ratio for 1% BP/BS (U40-20 min) was slightly greater than that for 1% BP/BS (U40-10 min) in 12 h, but was less than that for 1% BP/BS (U40-10 min) in 48 h. The results showed that, compared to 1% BP/BS without ultrasound, the ratios of isoflavone aglycones in the FE could be efficiently increased by the bioconversion of isoflavone glycosides, due to the enhanced β-glucosidase activities of *L. plantarum* FEL112 that were treated with ultrasound (40 or 120 kHz) for a relatively shorter time (10 min) (Figure 3). This also indicated that a suitable ultrasonic stimulation was beneficial for inducing higher β-glucosidase activity of *L. plantarum* FEL112 to produce fermented foods with a higher ratio of aglycones in their isoflavone content.

### 3.6. FE-SEM Images of L. plantarum FEL112

**Figure 5:**
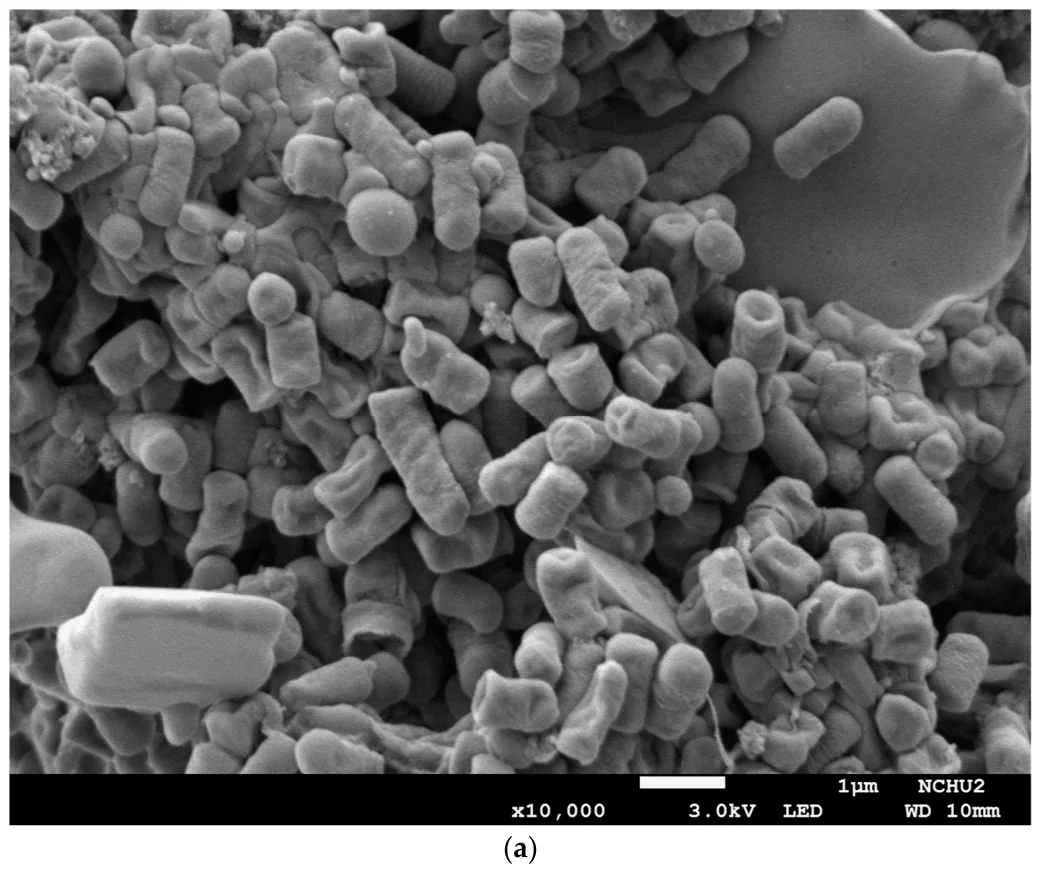

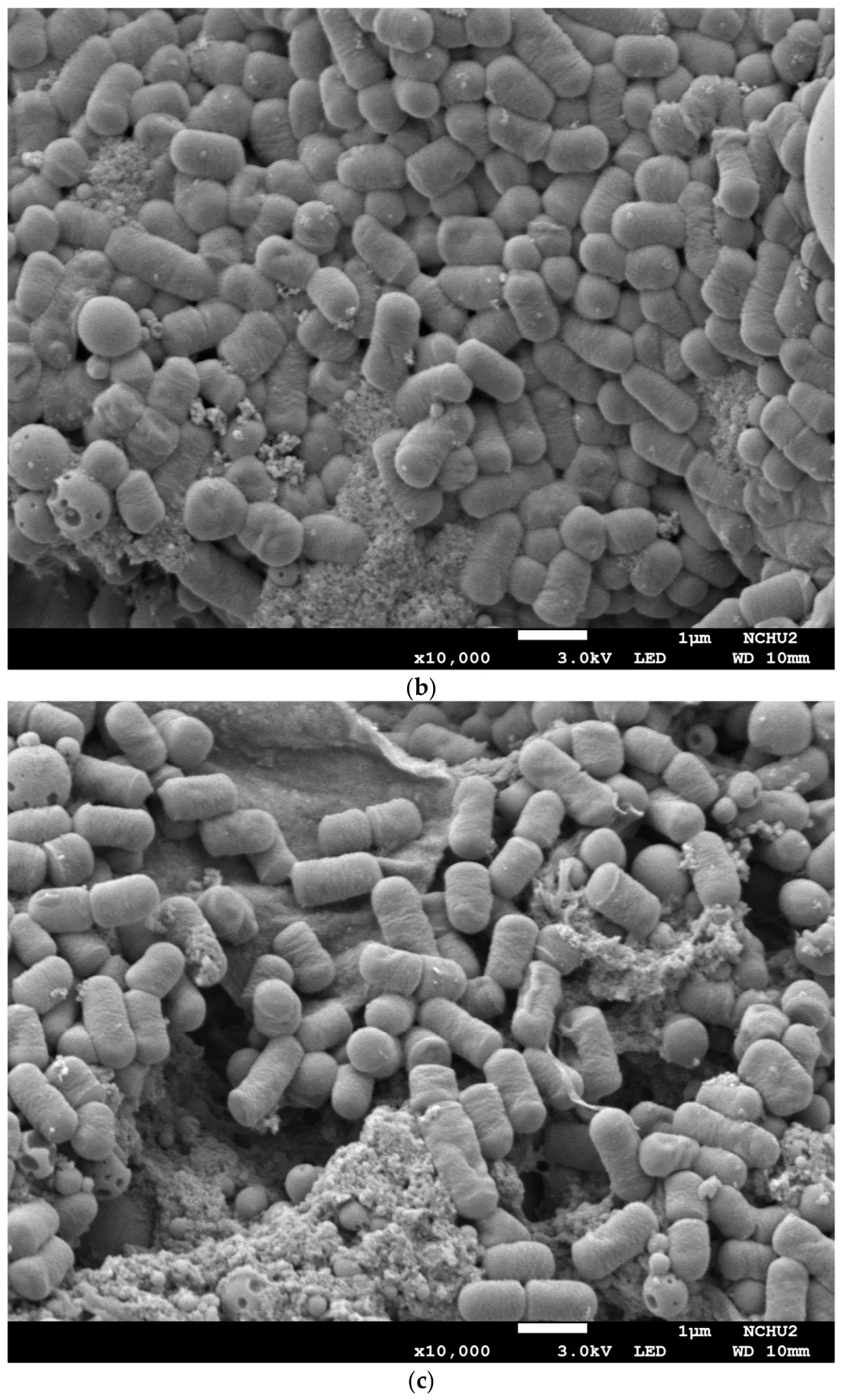
The FE-SEM images of the microstructure of *L. plantarum* FEL112 in 24 h of fermentation at 37 °C for (**a**) 1% BP/BS, (**b**) 1% BP/BS (U40-10 min), and (**c**) 1% BP/BS (U120-10 min). 1% BP/BS: the medium of 1% freeze-dried burdock root powders (BP) added in black soymilk (BS); 1% BP/BS (Ux-y min): the 1% BP/BS medium with ultrasonic treatment using x kHz of ultrasound for y min.

The FE-SEM images (×10,000) of the microstructure of *L. plantarum* FEL112 at 24 h of fermentation time at 37 °C for 1% BP/BS, 1% BP/BS (U40-10 min), and 1% BP/BS (U120-10 min) are shown in Figure 5a–c.

As shown in Figure 5a for 1% BP/BS without ultrasound, the surface of *L. plantarum* FEL112 was not very smooth but had a lot of wrinkles and sunken portions. When a higher intensity of ultrasound of 40 kHz was introduced to stimulate the strain in the medium for 10 min, the surface morphology of *L. plantarum* FEL112 was observed to be smoother after 24 h of incubation, as shown in Figure 5b for 1% BP/BS (U40-10 min). Figure 5c presents the surface morphology of *L. plantarum* FEL112 for 1% BP/BS (U120-10 min). When encountering ultrasonic stimulation with a lower intensity of 120 kHz of ultrasound for 10 min, the surface of *L. plantarum* FEL112 at 24 h was smooth but still had some slightly sunken portions. This indicated that the morphology of bacteria after a period of incubation could be affected by ultrasonic intensity, incubation time, and the nutritional composition of the medium.

## 4. General Discussion

This study demonstrated the impacts of BP added as a supplementary nutritional source and ultrasonic treatment before incubation on the growth and β-glucosidase activity of *L. plantarum* FEL112 in the fermentation of BS. Without using ultrasonic treatment for various amounts of BP in BS media, higher viable cell counts in 3% BP/BS than in BS (control), 1% BP/BS, and 7% BP/BS (Figure 1a), along with a lower pH value for 3% BP/BS than for other media, were observed (Figure 1b). A more acidic surrounding medium would substantially reduce the release of enzymes and inhibit the activity of β-glucosidase. In addition, the higher amount of solid BP with a network structure added to the BS medium, the larger the steric hindrance for *L. plantarum* FEL112 to utilize the available nutrients would be, so as to affect the growth of the strain. Gustaw et al. (2021) indicated that *L. plantarum* is the species known for its adaptations to a variety of environments, including fermented foods, and each *L. plantarum* strain has a unique repertoire of genes for the transport and utilization of sugars, corresponding to the carbohydrate composition of a fresh or fermented vegetable or fruit [27]. This suggested that the growth behavior of *L. plantarum* FEL112 in the BS could be altered by regulating the supplementary nutrients from solid BP in the medium environment along with the changes in the expression of β-glucosidase activity.

The results for various amounts of BP added in BS showed that the β-glucosidase activity of the strain in the medium of 1% solid BP in BS (1% BP/BS) was higher than those in BS (control), 3% BP/BS, and 7% BP/BS (Figure 2). It should be noted that the β-glucosidase activity of *L. plantarum* is highly adaptable, and can be modulated by specific nutrients existing in the culture medium, as indicated by Paventi et al. (2026) [28]. Since the solid BP with a network structure contained a lot of the polysaccharide inulin, which has the potential to bind with caffeoylquinic acids to form a complex during processing [19], the nutritional effect in the BS medium could be substantially altered by the addition of various amounts of BP. Moreover, the steric hindrance raised from the addition of solid BP in BS could lead to the situation that the contact and utilization efficiency of the nutrients from BP by *L. plantarum* FEL112 would be more impeded in 3% BP/BS than in 1% BP/BS, as reflected in the expression of β-glucosidase activity [22]. In addition, the steric hindrance for the strain to utilize the nutrients and the release of β-glucosidase could be much more impeded in the 7% BP/BS medium than in the 1%/BP/BS and 3% BP/BS media, leading to a rather low β-glucosidase activity. The results suggested that to improve the efficiency of enzymatic hydrolysis conducted during the fermentation of BS, the level of β-glucosidase activity was better considered as the indicator to select 1% BP as the supplementary nutrients in the BS medium to further investigate the impact of ultrasonic treatment on the fermentation system.

Ultrasound technology has been applied in food processes used either as a pre-treatment or during the operation to improve process efficiency, including extraction, freezing, drying, microbial safety, and fermentation, and is also employed as an effective means in the homogenization of solid–liquid mixtures [29]. In the fermentation process, the acoustic cavitation and microstreaming induced during the ultrasonic treatment alter the quality of the fermented products and enhance the activity of extracellular enzymes [29,30]. Shokri et al. (2020) indicated that *Lactococcus lactis* subsp. *Lactis* (PTCC 1336) was pretreated by ultrasound at a frequency of 24 kHz for 5 min; after ultrasonic stimulation, the cells were incubated in a nutrient broth at 30 °C, and the viable cell count was 25.35% higher than that without ultrasonic pretreatment [31]. Moreover, moderate and high intensity of ultrasonic treatments have different roles in *LactoBacillus brevis* activity [29]. In this study, the ultrasonic treatment using either 40 or 120 kHz for 10 or 20 min on the BS medium that contained 1% solid BP after inoculation of the strain and before starting fermentation, resulted in microbial proliferation with the viable cell counts that were all significantly higher than those without ultrasound after 12 h of incubation time, and the difference in the intensity of ultrasonic frequency (40 or 120 kHz) also induced different growth behaviours in the strain (Table 1). The results for applying ultrasonic treatment in the present microbial system exhibited similar findings in the viable cell counts as the performance of *Lactococcus lactis* subsp. *Lactis* (PTCC 1336) and *L. plantarum* AF1 [30]. In this study, the application of ultrasonic treatment on the 1% BP/BS fermentation system could also improve the homogenization of solid BP in the BS medium and help loosen the network structure of solid BP, so that the mass transport of ingredients and contact of the strain to the available nutrients in the fermentation system could be enhanced.

With ultrasonic treatment, the enzyme activities can be either promoted or inhibited. In this study, with ultrasonic treatment using either 40 or 120 kHz for the 1% BP/BS medium, the β-glucosidase activities were all significantly higher than those without ultrasonic treatment during 48 h of incubation (Figure 3). This finding was similar to those reported for the enhanced β-galactosidase activity of *Lactococcus lactis* subsp. *Lactis* stimulated with 24 kHz of ultrasound for 5 min after 12.5 h incubation by Shokri et al. (2020) [31] and for the significantly higher β-galactosidase activity of *L. plantarum* AF1 pre-treated with 30 kHz of ultrasound for 15 min compared to the control by Gholamhosseinpour and Hashemi (2019) [32]. Furthermore, in the present study with ultrasonic treatment on 1% BP/BS, the incubation time for the highest β-glucosidase activity of the strain to occur differed with varying ultrasonic frequencies and treatment times. Behzadnia et al. (2019) also concluded that the microbial activity and the productivity of biological processes could be intensified by controlled ultrasound exposure and supplementation of culture media [23]. It was speculated that, when using different intensities of ultrasonic stimulation on *L. plantarum* FEL112 for different treating times, the time needed for cell recovery from injury could be different and reflected in the expression of β-glucosidase activity during the fermentation [13,23].

Ultrasonic treatment could improve the texture of the fermented substrate, thereby affecting the growth of microorganisms and enzyme activity, resulting in changes in the functionality of the fermented product. Yu et al. (2021) indicated that the promoted enzyme activity was possibly attributed to the collective effects of various conditions of ultrasonic treatment [30]. Imtiaz et al. (2026) also reported that ultrasound-driven co-culture fermentation with lactic acid bacteria significantly enhanced the bioactive moieties’ composition and antioxidant capacity of Chinese yam [33]. In the optimized treatment group with ultrasound-assisted fermentation by *L. plantarum* and *Lactobacillus helveticus*, a higher TPC value (8109.74 µg/mL) was obtained, and FRAP enhanced significantly [33]. In the present study, for the effect of fermentation with or without ultrasonic stimulation on TPC and FRAP in FE (Figure 4), it was demonstrated that ultrasonic treatment could enhance the TPC and antioxidant activity of FE during the fermentation process. Among different ultrasonic treatment conditions, the ultrasonic stimulation with higher intensity (40 kHz) for a shorter time (10 min) is beneficial for improving the TPC and antioxidant activity of the fermented product of BP in BS.

Isoflavones are an important component for evaluating the fermentation process of soymilk, and isoflavone glycosides are hydrolyzed to their aglycones during fermentation [11]. As shown in the ratios of isoflavone aglycones in the FE (Table 2), whether using 40 or 120 kHz ultrasonic treatment, it would be beneficial for improving the AI/TI ratio. It was speculated that ultrasonic treatment enhanced β-glucosidase activities of *L. plantarum* FEL112 (Figure 3), and loosened the network structure of solid BP due to the cavitation effect of ultrasound, leading to the improvement in the bioconversion of isoflavones. Similar phenomena were also found in the fermentation of BS containing BWE in our previous study, the fractions of AI in the TI of the fermented product for ultrasonic stimulation were better than those without ultrasound [18]. Yeo and Liong (2011) indicated the enhancement of the intracellular and extracellular β-glucosidase activity of probiotics by using ultrasonic treatment at 100 W for 2 and 3 min, thereby increasing the bioconversion of glucosides to aglycones in prebiotic soymilk [13].

The FE-SEM images of *L. plantarum* FEL112 in the fermentation of 1% BP/BS with or without ultrasonic treatment are shown in Figure 5. It indicated that the morphology of bacteria could be affected by ultrasonic intensity, incubation time, and the nutritional composition of the medium. Liu et al. (2024) explored the effects of ultrasonic pretreatment on the fermentation performance and indicated that using ultrasound of 360 W at a frequency of 40 kHz for 5 min, the cell membrane of lactic acid bacteria produced repairable damage and the morphology did not significantly change [34].

As discussed above, the nutrient sources in the culture environment and ultrasonic treatment significantly affected the growth of the strain and its expression of β-glucosidase activity during the fermentation. In practice, the nutrient sources in the culture medium obtained by adding the aqueous extract of burdock root (BWE) to BS were substantially different from those obtained by using BP in BS. As compared with our previous study [18], the similar enhancement in the growth and β-glucosidase activity was achieved by using BP to replace BWE in the fermentation of BS combined with ultrasonic treatment in this study. This suggested that saving the pre-extraction step needed to obtain BWE could be possible for improving the economic benefits of the fermentation process by using a proper amount of BP added in BS.

## 5. Conclusions

In this study, the impacts of BP added in BS and ultrasonic treatment on the growth and β-glucosidase activity of *L. plantarum* FEL112 in the fermentation of BS were investigated. Without ultrasonic treatment, using the 1% BP/BS medium could obtain the highest β-glucosidase activity during 48 h of incubation among the tested media with different amounts of BP. Different frequencies of ultrasonic stimulation employed for 10 or 20 min before fermentation in 1% BP/BS exhibited various levels of enhancement in the β-glucosidase activity of *L. plantarum* FEL112. The antioxidant activities of FRAP, TPC, and the isoflavone aglycones ratio in the FE of 1% BP/BS were all enhanced with various frequencies of ultrasonic treatment. Compared with our previous study using an aqueous extract of burdock root added to BS combined with ultrasonic treatment [18], using BP instead of burdock root extract added to BS combined with ultrasonic treatment in the fermentation of BS by *Lp*-FEL112 could have the economic benefits by saving the extraction step needed to retain the full nutrient compounds of burdock root while achieving the same purpose of enhancement in the functionality of FE, exhibiting potential to be used in fermented foods.

## Figures and Tables

**Table 1:** Effect of various ultrasonic frequencies and treatment times on viable cell counts of *L. plantarum* FEL112 incubated in the medium of 1% BP/BS *.

Incubation Time (h)	Viable Cell Counts (log CFU/mL) in the Medium				
	1% BP/BS	1% BP/BS (U40-10 min)	1% BP/BS (U40-20 min)	1% BP/BS (U120-10 min)	1% BP/BS (U120-20 min)
0	6.59 ± 0.12 ^ab^	6.57 ± 0.06 ^b^	6.81 ± 0.16 ^a^	6.60 ± 0.04 ^ab^	6.68 ± 0.17 ^ab^
12	8.83 ± 0.08 ^b^	9.00 ± 0.07 ^a^	8.87 ± 0.11 ^ab^	8.98 ± 0.04 ^ab^	8.98 ± 0.08 ^a^
24	8.81 ± 0.02 ^b^	8.95 ± 0.02 ^a^	8.96 ± 0.08 ^a^	9.02 ± 0.08 ^a^	8.81 ± 0.04 ^b^
36	8.80 ± 0.01 ^b^	8.86 ± 0.02 ^ab^	8.87 ± 0.19 ^ab^	9.04 ± 0.15 ^a^	8.70 ± 0.10 ^b^
48	8.73 ± 0.05 ^c^	8.87 ± 0.09 ^b^	8.93 ± 0.05 ^ab^	9.03 ± 0.08 ^a^	8.71 ± 0.03 ^c^

* Conditions: incubation at 37 °C for 48 h. 1% BP/BS: the medium of 1% freeze-dried burdock root powders (BP) added in black soymilk (BS); 1% BP/BS (Ux-y min): the 1% BP/BS medium with ultrasonic treatment using x kHz of ultrasound for y min. Data were expressed as mean ± standard deviation (*n* = 3). Different superscript letters at the same incubation time were significantly different (*p* < 0.05) by Duncan’s multiple range test.

**Table 2:** Comparison of isoflavone profile in the dried fermented product extract (FE) of 1% BP/BS by *L. plantarum* FEL112 at 37 °C without or with various conditions of ultrasonic treatment *.

Medium Condition	Fermentation Time (h)	Isoflavones (μg/g-FE)				AI/TI (%)
		Daidzin	Genistin	Daidzein	Genistein	
1% BP/BS	12	32.81 ± 7.64	103.30 ± 23.24	139.43 ± 37.86	93.13 ± 22.82	63.08
	48	31.63 ± 3.30	72.28 ± 4.72	181.53 ± 18.35	121.72 ± 17.31	74.48
1% BP/BS (U40-10 min)	12	44.93 ± 2.57	42.60 ± 0.73	167.29 ± 0.78	112.53 ± 0.66	76.17
	48	47.27 ± 7.39	29.40 ± 0.27	204.92 ± 3.85	136.72 ± 3.51	81.67
1% BP/BS (U40-20 min)	12	38.34 ± 12.43	39.90 ± 2.87	160.09 ± 7.20	107.13 ± 4.74	77.35
	48	43.02 ± 4.19	38.65 ± 0.69	182.49 ± 6.93	128.26 ± 5.91	79.19
1% BP/BS (U120-10 min)	12	44.02 ± 8.50	37.75 ± 1.63	188.88 ± 13.47	126.75 ± 9.11	79.42
	48	37.91 ± 4.69	32.51 ± 4.53	188.43 ± 16.51	127.87 ± 7.23	81.79
1% BP/BS (U120-20 min)	12	47.42 ± 2.56	49.95 ± 2.67	166.78 ± 8.24	110.98 ± 6.06	74.04
	48	47.89 ± 1.17	29.62 ± 1.71	192.43 ± 14.71	133.50 ± 8.89	80.79

* Data were expressed as mean ± standard deviation (*n* = 3). 1% BP/BS: the medium of 1% freeze-dried burdock root powders (BP) added in black soymilk (BS); 1% BP/BS (Ux-y min): the 1% BP/BS medium with ultrasonic treatment using x kHz of ultrasound for y min. AI = sum of daidzein and genistein, TI = sum of daidzin, genistin, daidzein, and genistein. (AI/TI) (%) = (AI/TI) × 100%.

## Data Availability

The original contributions presented in this study are included in the article. Further inquiries can be directed to the corresponding author.
